# Napabucasin Induces Mouse Bone Loss by Impairing Bone Formation via STAT3

**DOI:** 10.3389/fcell.2021.648866

**Published:** 2021-03-18

**Authors:** Xiangru Huang, Anting Jin, Xijun Wang, Xin Gao, Hongyuan Xu, Miri Chung, Qinggang Dai, Yiling Yang, Lingyong Jiang

**Affiliations:** ^1^Shanghai Key Laboratory of Stomatology & Shanghai Research Institute of Stomatology, Department of Oral and Cranio-maxillofacial Surgery, National Clinical Research Center of Stomatology, Center of Craniofacial Orthodontics, Ninth People’s Hospital, Shanghai Jiao Tong University School of Medicine, Shanghai, China; ^2^Shanghai Key Laboratory of Stomatology & Shanghai Research Institute of Stomatology, National Clinical Research Center of Stomatology, The 2nd Dental Center, Ninth People’s Hospital, Shanghai Jiao Tong University School of Medicine, Shanghai, China

**Keywords:** napabucasin, STAT3, BMSCs, osteopenia, osteogenesis

## Abstract

The novel small molecule Napabucasin (also known as BBI608) was shown to inhibit gene transcription driven by Signal Transducer and Activator of Transcription 3 (STAT3), which is considered a promising anticancer target. Many preclinical studies have been conducted in cancer patients examining the selective targeting of cancer stem cells by Napabucasin, but few studies have examined side effects of Napabucasin in the skeleton system. In the present study, we found treating bone marrow mesenchymal stem cells (BMSCs) with Napabucasin *in vitro* impaired their osteogenic differentiation. In terms of mechanisms, Napabucasin disrupted differentiation of BMSCs by inhibiting the transcription of osteogenic gene osteocalcin (Ocn) through STAT3. Moreover, through micro-CT analysis we found 4 weeks of Napabucasin injections induced mouse bone loss. Histological analysis revealed that Napabucasin-induced bone loss in mice was the result of impaired osteogenesis. In conclusion, this study provided evidence for the effect of Napabucasin on mouse bone homeostasis and revealed its underlying mechanisms *in vivo* and *in vitro*.

## Introduction

Signal Transducer and Activator of Transcription 3 (STAT3), which plays a role in cell proliferation and other significant functional activities, is a key regulator of many oncogenic pathways and is therefore considered a promising anticancer target (Hubbard and Grothey, 2017; Aigner et al., 2019). In recent years, scientists have paid more attention to the crosstalk between immune and skeletal systems transmitted via critical signals, including STAT3, which is a key oncogenic transcription factor and is also reported to be a significant signal for bone homeostasis (Li, 2013). Several STAT3 inhibitors have been shown to affect the survival and differentiation of osteoblasts and osteoclasts (Li et al., 2013, 2018; Cheng et al., 2017; Cong et al., 2017). Furthermore, it was reported that Icariin prevented estrogen deficiency–induced bone loss through the activation of STAT3 (Xu et al., 2020).

Napabucasin (also known as BBI608), the focus of this investigation, is a novel small molecule shown to inhibit gene transcription driven by STAT3 (Hubbard and Grothey, 2017). Based on the promising effects observed in animal xenograft models (Li et al., 2015; Zhang et al., 2016), several preclinical studies were conducted in patients with advanced or metastatic cancers (Li et al., 2015, 2020; Bekaii-Saab et al., 2017; Grothey et al., 2017; Nagaraju et al., 2020). In fact, when this manuscript was written, there were 27 preclinical studies listed in the ClinicalTrials.gov database. Encouragingly, BBI608 blocked survival and self-renewal of stemness-high cancer cells. A multicenter phase I/II trial assessed that Napabucasin with pembrolizumab treatment showed antitumor activity for patients with metastatic colorectal cancer (Kawazoe et al., 2020). Li et al. (2015) also found that Napabucasin selectively targets cancer stem cells without affecting normal hematopoietic stem cells.

Bone marrow mesenchymal stem cells (BMSCs) are the major stem cell for osteogenesis. According to our previous studies, STAT3 significantly regulated rodent bone anabolism and was an alternative molecular target for the treatment of bone diseases (Xu et al., 2020). Similarly, researchers reported that inactivation of STAT3 in osteoblasts disrupted bone formation (Itoh et al., 2006; Zhou et al., 2011). Nevertheless, in the reported pre-clinical studies about Napabucasin, the most frequently reported adverse events were grade 1–2 gastrointestinal toxicities, diarrhea, nausea, vomiting, abdominal cramps, and fatigue (Langleben et al., 2013; Bekaii-Saab et al., 2017; Bendell et al., 2017). Currently, to the best of our knowledge, no study has ever mentioned any effects in the skeletal system, and there is little evidence *in vitro* or *in vivo* on the effects of napabucasin in bone metabolism.

As the number of preclinical studies about Napabucasin in cancer treatment has increased, the latent side effects on bone metabolism should not be ignored. The present study examined the effects of Napabucasin on mouse bone homeostasis and determined its underlying mechanisms *in vivo* and *in vitro*. Our results suggest that monitoring bone mass may help improve the quality of life and lengthen the life spans of cancer patients treated by Napabucasin.

## Materials and Methods

### Cell Culture and Osteoblastic Differentiation

BMSCs were washed out from the femurs and tibias of 4-week-old wild-type (WT) C57BL/6 mice as previously described (Yang et al., 2019). The bone marrow was flushed with a 10-ml injection of α-MEM (Corning, Cat#: 10-022-CV). BMSCs were cultured in α-MEM with 10% fetal bovine serum (Gibco, Cat#: 10099-141) and 1% penicillin-streptomycin (Gibco, Cat#: 15140122) at 37°C in 5% CO_2_. The medium was refreshed every 3 days until BMSCs reached 70–80% confluence. The cells were passaged and seeded into six-well plates at a density of 1.0 × 10^5^ cells per well and treated with 0.01, 0.1, 1, or 2 μM Napabucasin (Selleck, Cat#: S7977), with 1 μL/mL DMSO (Cyagen, Cat#: MUBMX-90021) as control in osteogenic induction medium according to our previous study (Xu et al., 2020).

### CCK8 Assay

After BMSCs reached 80–90% confluence, they were treated with Napabucasin at the dose of 0.01, 0.1, 1, or 2 μM, while 1 μL/mL DMSO was used for control cells. A CCK8 kit (Beyotime, Cat#: C0037) was used to measure cell proliferation and survival of treated BMSCs on days 1, 3, 5, and 7 following the manufacturer’s protocol.

### Annexin V FITC Apoptosis Assay

An Annexin V FITC Apoptosis Detection Kit (Beyotime, Cat#: C1062S) was used to assess the effect of Napabucasin on apoptosis in BMSCs. After BMSCs reached 80–90% confluence, they were treated with Napabucasin at the dose of 0.01, 0.1, 1, or 2 μM for 2 days according to previous study (Xu et al., 2020), while DMSO was used as a control. Cells were collected and analyzed using the FACSCalibur system (BD Biosciences).

### Western Blot Assay

Cells were lysed with SDS lysis buffer (Beyotime, Cat#: P0013G) and mixed with protease and phosphatase inhibitors (Thermo Fisher Scientific). Western blot assays were performed using a previously described protocol (Yang et al., 2019). The primary antibodies used were as follows: β-actin (Cell Signaling Technology, Cat#: 3700), STAT3 (Cell Signaling Technology, Cat#: 9139), and pSTAT3 (Cell Signaling Technology, Cat#: 9145). After incubation, the membranes were washed and incubated in HRP-linked IgG secondary antibody (Beyotime, Cat#: A0239, A0258). An enhanced chemiluminescence detection system was used to visualize the protein.

### Alkaline Phosphatase and Alizarin Red Staining

BMSCs were fixed in 4% paraformaldehyde at room temperature for 10 min and stained with alkaline phosphatase (ALP) working solution (Beyotime) at 37°C in the dark for 2 h or with 40 mmol/L Alizarin Red (Cyagen, Cat#: MUBMX-90021) for 15 min following the manufacturer’s protocol.

### RT-PCR Assay

RNA was extracted using TRIzol reagent (Takara, Cat#: 9109) and reverse-transcribed into cDNA using a Prime Script RT master kit (Takara, Cat#: RR036A). The primers used were as follows. β-Actin sense: 5′-CCCATACCCACCATCACACC-3′, β-Actin antisense: 5′-CACCCGCGAGTACAACCTTC-3′; Runx2 sense: 5′-CCTCCAGCATCCCTTTCTT-3′, Runx2 antisense: 5′-CCTCCAGCATCCCTTTCTT-3′; Col1a1 sense: 5′-GCTCCTCTTAGGGGCCACT-3′, Col1a1 antisense: 5′-CCACGTCTCACCATTGGGG-3′; osteocalcin (Ocn) sense: 5′-GAATAGACTCCGGCGCTACC-3′, Ocn antisense: 5′-AG CTCGTCACAATTGGGGTT-3′; Alp sense: 5′-CGGGACTGG TACTCGGATAA-3′, Alp antisense: 5′-ATTCCACGTCGGTT CTGTTC-3′.

### Luciferase Assay

HEK 293T cells were seeded into 24-well plates. Plasmids containing an Ocn promoter-driven pGL3-based luciferase reporter gene, along with plasmids encoding Stat3 and Renilla luciferase, were transfected into cells with Lipofectamine 2000 (Thermo Fisher Scientific, Cat#: 11668019) according to a previously reported protocol (Xu et al., 2020). Cells were treated with different concentrations of Napabucasin (0.1, 1, or 10 nM). At 36–48 h after transfection, cells were collected and lysed and the supernatants were used for dual-luciferase reporter assays according to the manufacturer’s instructions (Promega, Cat#: E1960).

### Cleavage Under Targets and Tagmentation (CUT&Tag) and Quantitative RT-PCR Analysis

The CUT&Tag assay was performed with the Hyperactive *In Situ* ChIP Library Prep Kit for Illumina (Vazyme, Cat#: TD901-01) as previously described according to the manufacturer’s instructions (Kaya-Okur et al., 2019; Dan et al., 2020). Briefly, C3H10 T1/2 cells treated with 0.1 μM Napabucasin or vehicle control were washed with wash buffer containing 1× protease inhibitor cocktail (Sigma-Aldrich, Cat#: 5056489001). Cell pellets were resuspended in wash buffer and Concanavalin A-coated magnetic beads were added and incubated at room temperature. Bead-bound cells were resuspended in antibody buffer (20 mM HEPES pH 7.5, 150 mM NaCl, 0.5 mM spermidine, 0.05% digitonin, 2 mM EDTA, 0.1% BSA and 1× protease inhibitor cocktail). Then, 1 μg of STAT3 antibody (Cell Signaling Technology, Cat#: D3Z2G) or normal IgG (Cell Signaling Technology, Cat#: 2729) was added and incubated overnight at 4°C. After removing the primary antibody, 1 μg of secondary antibody (Vazyme, Cat#: ab206) diluted in Dig-wash buffer (20 mM HEPES pH 7.5, 150 mM NaCl, 0.5 mM spermidine, 0.05% digitonin and 1× protease inhibitor cocktail) was added and incubated at room temperature. The cells were then incubated with Hyperactive pG-Tn5 Transposase diluted in Dig-300 buffer (20 mM HEPES pH 7.5, 300 mM NaCl, 0.5 mM spermidine, 0.01% digitonin and 1× protease inhibitor cocktail) at room temperature for 1.5 h. Finally, the cells were resuspended in tagmentation buffer (10 mM MgCl_2_ in Dig-300 buffer) and incubated at 37°C for 1.5 h. DNA was purified using phenol-chloroform-isoamyl alcohol extraction and ethanol precipitation after RNase A treatment. Precipitated DNA was detected by quantitative RT-PCR with specific primers. The primers for the STAT3 binding site in the Ocn promoter were 5′GGATACCCCATGTTCCCAGC3′ and 5′TGCAGCCCGTCTACTGGAGC3′.

### Animals and Treatment

Non-pregnant female C57BL/6 mice (4 weeks old, *n* = 22) were purchased from Charles River Laboratories (Shanghai, China). All mice were bred and maintained under specific pathogen-free conditions in a temperature-controlled room (21°C) with a 12 h light/12 h dark cycle and were provided food and water *ad libitum*. Mice were randomly divided into two groups as follows: Control group received injections of 5% DMSO + 40% PEG 300 + 5% Tween 80 + ddH2O (i.p.) every 2 days for 1 month; Napabucasin group received injection of Napabucasin (Selleck, Cat#: S7977; 10 mg/kg, i.p.) every 2 days for 1 month according to previous studies (Guha et al., 2019).

Ten mice were used for micro-CT analysis and were randomly divided into two groups: Control group (*n* = 5) and Napabucasin group (*n* = 5). Six mice were used for histological investigation and Alizarin red and calcein double labeling, and were divided into two groups: Control group (*n* = 3) and Napabucasin group (*n* = 3). Six mice were used for cell culture, *n* = 3 each for the control and Napabucasin groups.

All animal experiments were performed according to protocols approved by the animal care committee guidelines of the Shanghai Jiao Tong University biomedical ethics committee for laboratory animal welfare ethics.

### Micro-CT

Mice (8 weeks old) were euthanatized with carbon dioxide, and the femora were dissected and stored in ethanol, then scanned with a micro-CT scanner (Scanco Medical AG, Cat#: vivaCT 80). A 1-mm width of trabecular bone close to the distal growth plate of the femur and a 1-mm-wide section of cortical bone from the middle of the femur were reconstructed three-dimensionally and analyzed according to a previously described protocol (Yang et al., 2020). The microarchitectural parameters included in this experiment were bone mineral density (BMD), bone volume fraction (BV/TV), trabecular thickness (Tb.Th.), trabecular number (Tb.N.), trabecular separation (Tb.Sp.), and cortical thickness (Ct.Th.).

### Histological Analysis

Femurs from 8-week-old mice were fixed with 4% paraformaldehyde for 48 h. Specimens were then decalcified in 15% EDTA with an ultrasonic decalcifier for 3 weeks. Specimens were embedded in paraffin and cut into consecutive 4-μm sections. Paraffin sections were stained with hematoxylin and eosin according to a previously described protocol (Yang et al., 2020). Tartrate-resistant acid phosphatase (TRAP) staining was performed using a TRAP staining kit (MultiSciences, Cat#: 70-CK20203) according to the manufacturer’s protocol.

### Immunofluorescence Staining

Immunofluorescence staining was performed as described in a previous protocol (Zou et al., 2013). Sections were de-waxed and rehydrated, followed by antigen retrieval with proteinase K at 37°C for 20 min. Sections were blocked in PBS with 10% horse serum for 1 h and then incubated overnight at 4°C with antibodies against pSTAT3 (Cell Signaling Technology, Cat#: 9145), OCN (Santa Cruz Biotechnology, Cat#: sc-390877) and cathepsin K (CTSK; Santa Cruz Biotechnology, Cat#: sc-48353). Goat anti-mouse cy3 (Molecular Probes, Cat#: M30010) and goat anti-rabbit cy3 (Molecular Probes, Cat#: A10520) were used as secondary antibodies for 1 h at room temperature. DAPI (Sigma, Cat#: D8417) was used for counterstaining. Sections were mounted on slides with anti-fluorescence mounting medium (Dako, Cat#: S3023), and images were acquired with a confocal microscope (Leica, Cat#: Leica TCS SP8). The number of positively stained cells was counted in the whole femur subchondral bone area in each specimen according to previous study (Cui et al., 2016).

### Alizarin Red and Calcein Double Labeling

Mice received injections of calcein (Sigma, Cat#: C0875) and Alizarin Red (Sigma, Cat#: A5533) 7 and 3 days before euthanasia, respectively. Isolated femurs and tibias were dehydrated and embedded in polymethylmethacrylate. Specimens then were cut into continuous 5-μm thick sections with a rotary microtome. Images were captured as previously described (Yang et al., 2020).

### Statistical Analysis

All quantitative data were expressed as means ± S.D. Two groups were compared using independent-samples *t*-tests. One-way analysis of variance was performed for multiple comparisons. P < 0.05 was considered a significant difference. Data analysis was performed with SPSS 16.0 analysis software.

## Results

### Effects of Napabucasin on Proliferation and Apoptosis of BMSCs

**FIGURE 1:**
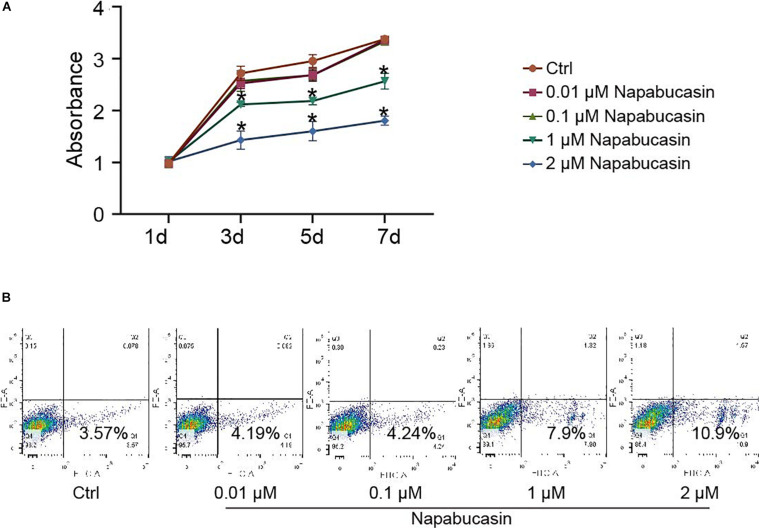
Effects of Napabucasin on proliferation and apoptosis of BMSCs. **(A)** CCK8 assay of BMSCs after treatment with DMSO or with different concentrations of Napabucasin for 1, 3, 5, and 7 days. *N* = 5. Data are represented as means ± S.D. *P < 0.05. **(B)** Apoptosis assay of BMSCs treated with DMSO or with different concentrations of Napabucasin for 2 days.

To investigate the effects of Napabucasin on BMSCs, we first isolated BMSCs from 4-week-old WT mice and cultured them with osteoblast differentiation medium, then treated them with different concentrations of Napabucasin to test their rates of proliferation and apoptosis. The growth curve of BMSCs was determined by CCK8 assay. As shown in Figure 1A, high doses of Napabucasin (1 or 2 μM) slowed BMSC proliferation in a dose-dependent manner. Furthermore, apoptosis assays of BMSCs after 2 days of Napabucasin treatment showed a concentration-dependent increase in the rate of apoptosis, especially at doses of 1 or 2 μM (Figure 1B). Therefore, lower concentrations of Napabucasin (0.01 or 0.1 μM) were used in the following experiments to explore the effects of Napabucasin on osteogenesis of BMSCs.

### Napabucasin Impaired Osteogenic Differentiation of BMSCs

**FIGURE 2:**
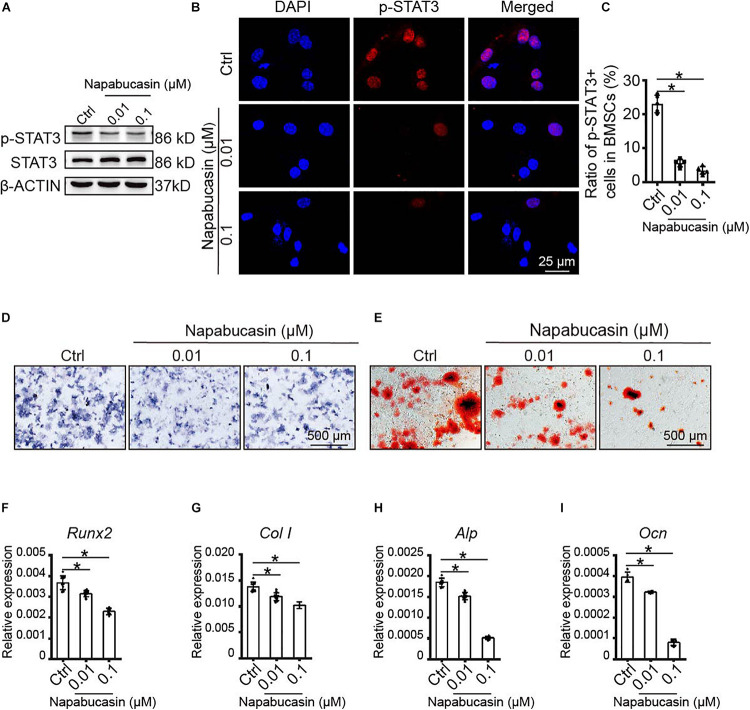
Napabucasin impaired osteoblast differentiation of WT BMSCs. **(A)** Western blotting assay of STAT3 and pSTAT3 expression in BMSCs treated with DMSO or with different concentrations of Napabucasin for 12 h. **(B)** Representative immunofluorescence image of pSTAT3 expression in BMSCs after exposure to Napabucasin. **(C)** Number of pSTAT3^+^ cells were counted. *N* = 5. **(D)** ALP staining of BMSCs after osteoblast differentiation for 7 days following treatment with DMSO or with different concentrations of Napabucasin. **(E)** Alizarin Red staining of BMSCs after osteoblast differentiation 14 days following treatment with DMSO or with different concentrations of Napabucasin. **(F–I)** Osteoblast-specific expression of genes *Runx2*, *Col I*, *Alp*, *and Ocn* in BMSCs during osteoblast differentiation after treatment with DMSO or with different concentrations of Napabucasin for 7 days. *N* = 9. Data are represented as means ± S.D. *P < 0.05.

Subsequently, we probed the protein expression of STAT3 and pSTAT3 in BMSCs treated with Napabucasin at the concentrations of 0.01 or 0.1 μM. Western blotting assay (Figure 2A) and immunofluorescence staining (Figures 2B,C) showed that Napabucasin inhibited pSTAT3 expression but not STAT3 in BMSCs after 12 h. We then examined the effects on osteogenic differentiation of BMSCs caused by Napabucasin treatment. ALP staining on day 7 of BMSC treatment with Napabucasin showed diminished ALP activity at Napabucasin concentrations of 0.01 or 0.1 μM compared with the control group (Figure 2D). Moreover, Alizarin Red staining on day 14 demonstrated fewer calcified nodules with Napabucasin at concentrations of 0.01 or 0.1 μM compared with the control group (Figure 2E). Quantitative RT-PCR showed downregulated expression of the osteogenic marker genes *Runx2*, *Col I*, *Alp*, and *osteocalcin (Ocn)* (Figures 2F–I). All these data indicated that Napabucasin impaired osteogenic differentiation of BMSCs, but the mechanism underlying Napabucasin regulation of osteogenic differentiation of BMSCs was still not clear.

### Napabucasin Modulated *Ocn* Transcription Through STAT3

**FIGURE 3:**
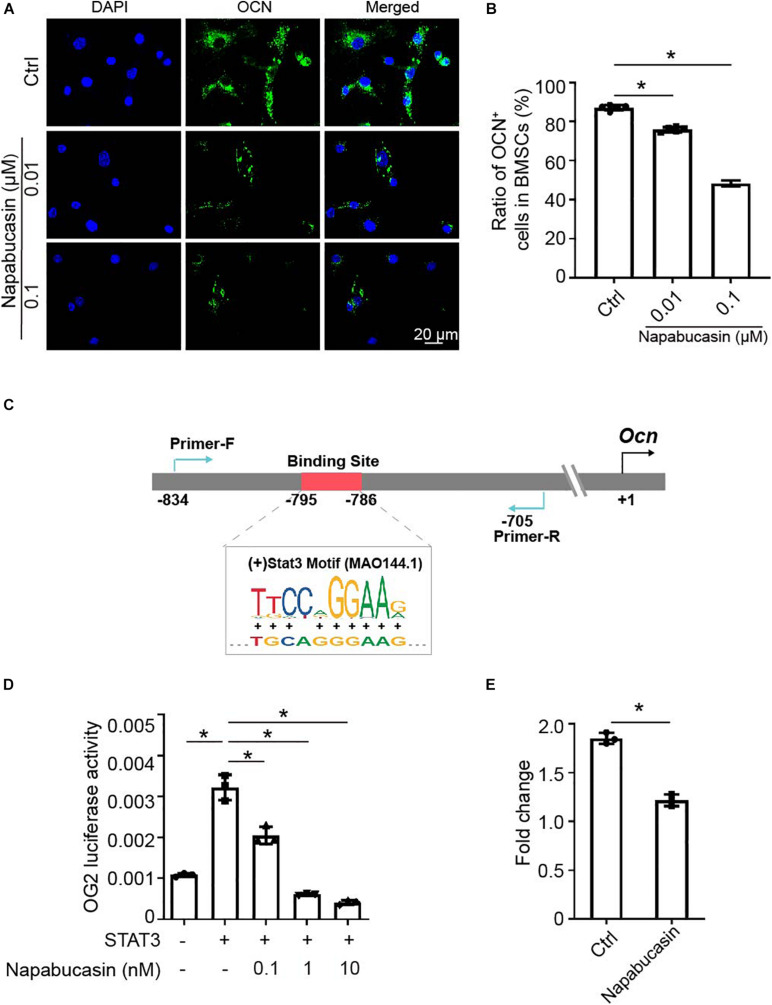
Napabucasin impaired *Ocn* transcription through STAT3. **(A)** Representative immunofluorescence image of OCN expression in BMSCs after exposure to Napabucasin. **(B)** Number of OCN^+^ cells were counted. *N* = 5. Data are represented as means ± S.D. **(C)** Illustration of predicted STAT3 binding sites in the *Ocn* promoter. **(D)** 293T cells were transfected with STAT3 and *Ocn* luciferase constructs and treated with DMSO or with different concentrations of Napabucasin. After 48 h, luminescent signals were detected to represent reporter activity. *N* = 5. Data are represented as means ± S.D. **(E)** CUT & Tag and subsequent quantitative PCR analysis of STAT3 showing decreased occupancy of the OCN promoter in C3H10 T1/2 cells treated with Napabucasin. Data are shown as fold enrichments to the IgG control. Data are represented as means ± S.D. *P < 0.05.

As Xu et al. reported previously, STAT3 regulated osteoblast differentiation of BMSCs by activating transcription of osteogenic gene *Ocn* (Xu et al., 2020). In this experiment, as shown in Figure 2I, the mRNA expression of *Ocn* was greatly downregulated after Napabucasin treatment. Immunofluorescence investigation of Napabucasin-treated BMSCs also showed decreased expression of OCN (Figures 3A,B). Therefore, in this experiment, we intended to test if STAT3-induced Ocn activation was the reason of impaired osteogenic differentiation under Napabucasin treatment. We then analyzed the promoter of *Ocn* and found one potential STAT3 binding site (Figure 3C). Next, we transfected promoter-driven luciferase reporters encoding *Ocn* and STAT3 into HEK 293T cells. Luciferase assays confirmed that STAT3 promoted *Ocn* transcription. Meanwhile, when we added Napabucasin to HEK 293T cells to block the function of STAT3, the enhancement of *Ocn* transcription was strongly inhibited (Figure 3D). The reduced binding of STAT3 to the *Ocn* promoter was validated by CUT & Tag-qPCR in C3H10 T1/2 cells treated with 0.1 μM Napabucasin (Figure 3E). Therefore, we assumed that Napabucasin modulated *Ocn* transcription through STAT3, eventually affecting osteogenic differentiation of BMSCs.

### Napabucasin Injection Caused Osteopenia in Mice

**FIGURE 4:**
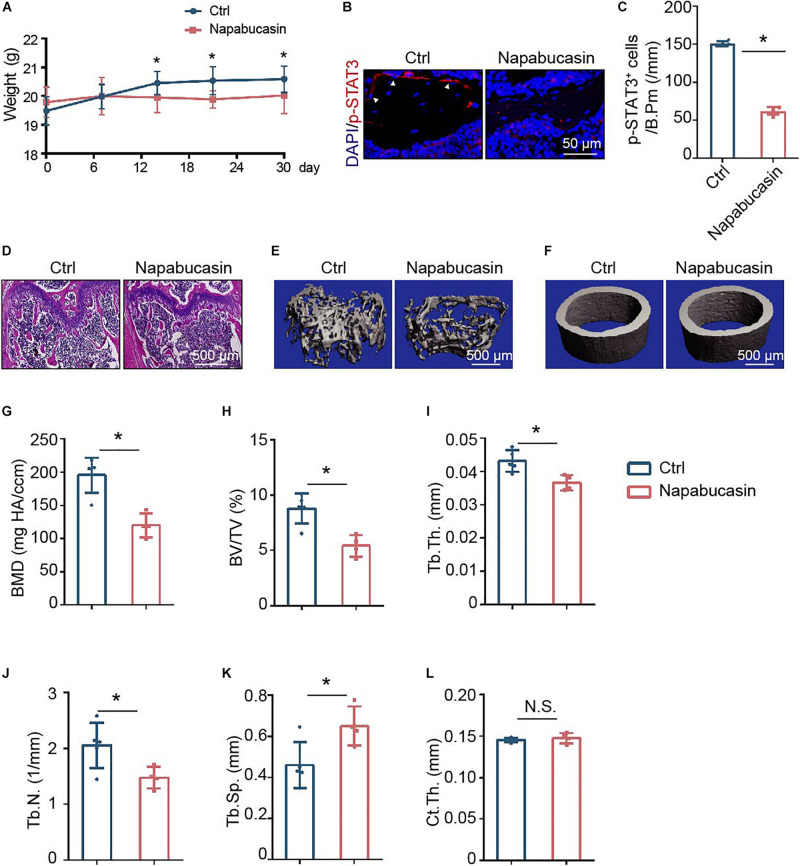
Napabucasin injection caused osteoporosis in mice. **(A)** Representative immunofluorescence image of pSTAT3 staining in the femurs of mice injected with vehicle or Napabucasin (10 mg/kg). **(B)** Number of pSTAT3^+^ cells were counted. *N* = 5. Data are represented as means ± S.D. *P < 0.05. **(C)** Body weights of mice injected with vehicle or Napabucasin (10 mg/kg) were recorded on different days. **(D,E)** Three-dimensional micro-CT reconstruction images of trabecular bone **(D)** and cortical bone **(E)** of femora from the above mice. **(F–K)** Quantitative microarchitectural parameters from micro-CT. *N* = 5. Data are represented as means ± S.D. *P < 0.05. **(L)** HE staining of femora from the above mice.

To directly investigate the *in vivo* effect of Napabucasin on bone metabolism, we injected Napabucasin into 4-week-old female mice. In our experiment, after injecting Napabucasin (10 mg/kg) for 1 month, the terminal mouse body weights were greatly reduced in the Napabucasin group compared with mice in the control group (Figure 4A). As shown in Figures 4B,C, the phosphorylation of STAT3 was obviously blocked after one-month of Napabucasin injections in our immunofluorescence investigation. H&E staining shown that Napabucasin injection-induced bone loss in the femora (Figure 4D). Then we assessed the bone mass of femora from mice injected with vehicle or Napabucasin by micro-CT. The bone loss of trabecular bone in the Napabucasin-injected group was shown by reconstructed micro-CT-scanned images (Figure 4E), but the bone mass of cortical bone did not appear to be different (Figure 4F). Quantitative microarchitectural parameters were measured to analyze the quantity and quality of bone in mice receiving Napabucasin injection or vehicle. Parameters such as BMD (Figure 4G), BV/TV (Figure 4H), Tb.Th. (Figure 4I), and Tb.N. (Figure 4J) were apparently reduced after 1-month injections of Napabucasin, while Tb.Sp. (Figure 4K) increased. However, Ct.Th. (Figure 4L) did not differ in this experiment. These results implied that the STAT3 inhibitor Napabucasin decreased the bone mass of WT mice.

### Napabucasin Impaired Bone Formation Without Influencing Bone Resorption

**FIGURE 5:**
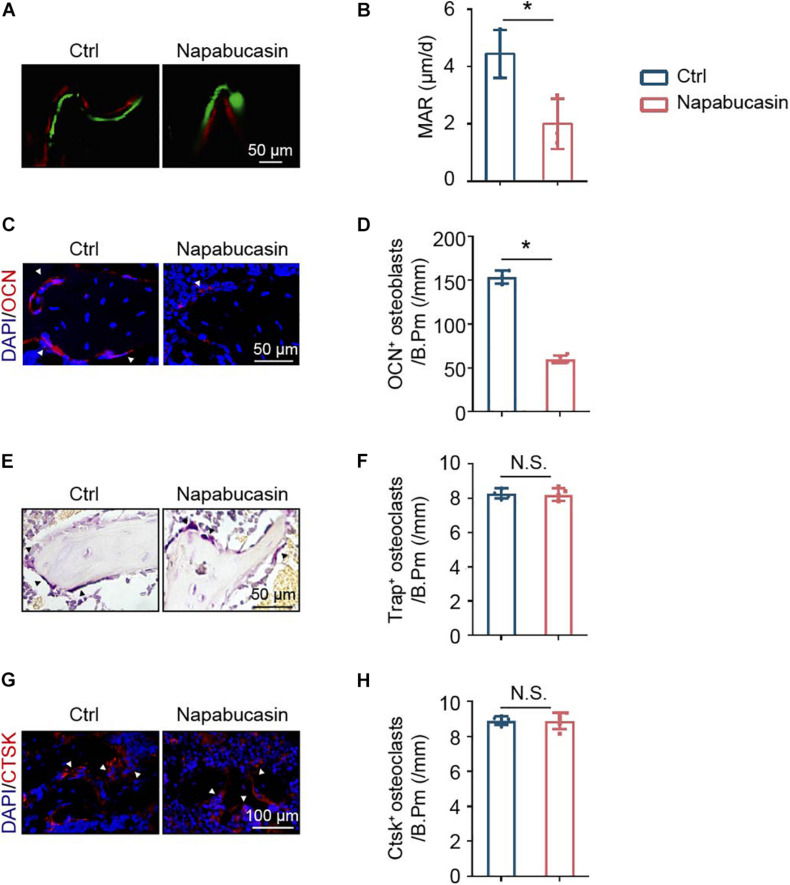
Napabucasin impaired bone formation but did not change bone resorption. **(A)** Osteogenic activity in femora of mice determined by calcein and Alizarin Red double staining. **(B)** Mineral apposition rate (MAR) detected by histomorphometric analysis. *N* = 3. **(C)** Representative immunofluorescence image of OCN staining in femurs from the above mice. **(D)** Number of OCN^+^ cells were counted. *N* = 5. **(E)** TRAP staining of femora from the above mice. **(F)** Number of TRAP^+^ multi-nucleated osteoclasts were counted. *N* = 5. **(G)** Representative immunofluorescence image of CTSK staining in the femurs from the above mice. **(H)** Number of CTSK^+^ cells were counted. *N* = 5. Data are represented as means ± S.D. *P < 0.05.

To explore the reason for bone loss caused by STAT3 inactivation *in vivo*, we inspected the bone metabolism of mice. Unbalanced bone metabolism is usually the result of abnormal bone formation and/or bone resorption. The mineral apposition rate (MAR) was measured by calcein and Alizarin Red double labeling (Figure 5A), which represented the new bone formation rate. As shown, after 1 month of Napabucasin injections, the MAR of trabecular bone from tibiae apparently decreased, indicating that Napabucasin impaired bone mineralization in the mice (Figure 5B). The downregulated expression of osteogenic marker OCN in immunofluorescence staining also implied impaired osteogenesis (Figures 5C,D). TRAP staining was performed to detect the change in osteoclastogenesis in femora from Napabucasin-injected mice (Figure 5E). The number of TRAP^+^ multi-nucleated osteoclasts from femora was not significantly influenced by Napabucasin injections (Figure 5F). Moreover, the immunofluorescence investigation of CTSK expression was fully consistent with that of TRAP staining (Figures 5G,H). In conclusion, Napabucasin-induced bone loss after 1 month of injections was the result of impaired bone formation.

## Discussion

The promising novel STAT3 inhibitor Napabucasin has undergone several preclinical studies (Hubbard and Grothey, 2017; Li et al., 2020; Nagaraju et al., 2020). Considering that the classic STAT3 trigger point of the immune system was also a significant signal in bone homeostasis (Li, 2013; Yang et al., 2019), we had good reason to be concerned that long-time anti-tumor treatment with Napabucasin would also affect the skeletal system of patients, which has never been reported to our knowledge.

Considering that Napabucasin can selectively target cancer stem cells (Li et al., 2015), we first tested the effect of Napabucasin on BMSCs as the major stem cell responsible for osteogenesis. Through CCK−8 and apoptosis assays, we determined that the optimal concentration for the effect of Napabucasin on BMSCs was 0.01–0.1 μM. Downregulated osteogenic differentiation of BMSCs treated with Napabucasin was determined by ALP staining and Alizarin Red staining, complemented by qPCR analysis of osteogenic marker genes. All of these data indicated that Napabucasin impaired the differentiation of BMSCs, but the underlying mechanism remained unknown.

It is known that activated STAT3 is phosphorylated and translocates to the nucleus to bind target-gene promoter sequences (Bromberg, 2002). Numerous key molecular markers and genes responsible for cancer stem cell proliferation were found to be downregulated by Napabucasin treatment (Li et al., 2015). As for BMSCs, according to our previous study, STAT3 directly bound to the Ocn promoter and subsequently activated its transcription (Xu et al., 2020). Importantly, the STAT3-activated transcription of *Ocn* could be obstructed by pharmaceuticals. Immunofluorescence investigation and qPCR showed an obvious decrease of OCN expression in Napabucasin-treated BMSCs. Therefore, in this experiment, we analyzed the role of Napabucasin on *Ocn* promoter activity using a luciferase reporter system and our data showed that Napabucasin affected *Ocn* transcriptional activity through STAT3 signaling. Moreover, the reduced binding of STAT3 to the *Ocn* promoter was validated by CUT & Tag-qPCR in BMSCs treated with Napabucasin. Therefore, we assumed that Napabucasin modulated *Ocn* transcription through STAT3, eventually affecting osteogenic differentiation of BMSCs.

In the present study, we found that 4-week Napabucasin injections induced mouse bone loss as a result of impaired osteogenesis. Initially, micro-CT analysis confirmed the bone loss of Napabucasin-injected mice, but the change in bone metabolism required further investigation. Interleukin-6 (IL-6)/Janus kinase (JAK)-2/STAT3 signaling has been widely studied in anti-tumor therapy and participates in the differentiation of osteoblasts. STAT3 is the downstream gene of multiple drugs inhibiting IL-6 and JAK-2 (Duplomb et al., 2008; Jo et al., 2018). In addition, the chemical inhibition of STAT3 phosphorylation abolished Icaritin-induced increases in osteoblast proliferation and function (Lim et al., 2017). In the present experiment, decreased bone formation, which was detected by calcein and Alizarin Red double labeling, was also considered to be the main reason for the aforementioned bone loss. Regarding bone catabolism, the increase of Tyrosine (705) phosphorylation of STAT3 upon IL-6 stimulation was reported to lead to the formation of macrophages instead of osteoclasts (Cheng et al., 2017; Li et al., 2018). According to our previous studies, STAT3 participates in osteoclast differentiation and ablation of Stat3 in osteoclasts resulted in decreased bone resorption *in vivo* (Yang et al., 2019), but in the present experiment, bone resorption was obviously not influenced by Napabucasin injection.

Clinical data shows that osteopenia is often a long-term complication of anti-neoplastic treatment (Aisenberg et al., 1998). As a first-in-class cancer stemness inhibitor, the side effects of Napabucasin should not be underestimated. Our research focused on the side effects of Napabucasin on the skeletal system, which may be helpful for monitoring bone mass over time. Napabucasin is an oral drug, and further research should be undertaken using oral administration to provide results with greater clinical value.

In conclusion, our data has provided evidence of an effect of Napabucasin on mouse bone homeostasis and determined its underlying mechanisms *in vivo* and *in vitro* involve modulation of STAT3. The latent side effects on bone metabolism after long-time anti-tumor therapy with Napabucasin should therefore be considered before patient treatment decisions.

## Data Availability Statement

The datasets generated for this study can be found in the online repositories. The names of the repository/repositories and accession number(s) can be found in the article/supplementary material.

## Ethics Statement

The animal study was reviewed and approved by the Animal Experimental Ethical Inspection Shanghai Ninth People’s Hospital affiliated to Shanghai Jiao Tong University, School of Medicine.

## Author Contributions

XH, AJ, and XW designed the experiment. XH, AJ, XW, HX, MC, and XG collected and analyzed the data. LJ, YY, and QD contributed to the interpretation of the results and critical revision of the manuscript and approved the final version of the manuscript. All authors agreed to be accountable for the content of this work.

## Conflict of Interest

The authors declare that the research was conducted in the absence of any commercial or financial relationships that could be construed as a potential conflict of interest.
